# The influence of recipient’s pre-existing diabetes mellitus on outcomes of living donor kidney transplant

**DOI:** 10.12669/pjms.42.9.13651

**Published:** 2026-09

**Authors:** Hina Shahabuddin, Zareen Kiran, Muhammad Tassaduq Khan, Abdur Rasheed

**Affiliations:** 1 Hina Shahabuddin, FCPS. National Institute of Diabetes and Endocrinology, Dow University Hospital, Dow University of Health Sciences, Karachi, Pakistan; 2 Zareen Kiran, FCPS, FRCP. National Institute of Diabetes and Endocrinology, Dow University Hospital, Dow University of Health Sciences, Karachi, Pakistan; 3 Muhammad Tassaduq Khan, FCPS, FRCP. National Institute of Solid Organ and Tissue Transplantation, Dow University Hospital, Dow University of Health Sciences, Karachi, Pakistan; 4 Abdur Rasheed, PhD. School of Public Health, Dow University of Health Sciences, Karachi, Pakistan

**Keywords:** Diabetes mellitus, Survival, Living donor, Kidney transplantation

## Abstract

**Objective:**

To determine the influence of recipients’ pre-existing diabetes mellitus on the outcomes of living donor kidney transplantation.

**Methodology:**

This prospective observational cohort study included 202 patients who underwent kidney transplantation between September 2022 and December 2023 at Dow University Hospital, Pakistan, and were stratified into two equal groups: pre-existing diabetes and non-diabetes. Patients with new-onset diabetes after transplantation were excluded. Demographic and clinical data were collected. Outcomes, including one-year patient survival and post-transplant complications, were assessed and compared.

**Results:**

Complications were high in the pre-existing diabetes group (57.4% vs. 28.7%, p <0.001). Infections occurred in 46.5% of patients with pre-existing diabetes versus 22.8% non-DM (p <0.001). Post-transplant cardiovascular events were observed in 6.9% of patients in the pre-existing DM group, whereas none were observed in the non-DM group (p = 0.014). One-year patient survival was 88.1% in the pre-existing DM group versus 95% in the non-DM group (p = 0.075). Graft-related complications did not differ significantly between the groups. Multivariate analysis identified pre-transplant congestive heart failure (HR = 99.431, [95% CI 7.709–128.441, p <0.001]), pre-transplant peripheral vascular disease (HR =7.787, [95% CI 1.197–50.682, p = 0.032]), post-transplant cardiovascular events (HR = 7.839, [95% CI 1.423–43.198, p = 0.018]), and post-transplant FBS (HR = 1.023, [95% CI 1.001–1.046, p = 0.040]) as independent predictors of mortality.

**Conclusion:**

Diabetes did not influence one-year patient survival and graft-specific outcomes but led to frequent post-transplant complications. Pre- and post-transplant cardiovascular disease and elevated post-transplant FBS levels were associated with mortality. Therefore, structured pre-transplant cardiovascular assessment and optimization, ongoing post-transplant cardiovascular surveillance, and strict glycemic control are required.

## INTRODUCTION

Diabetic kidney disease is the predominant cause of end-stage renal disease (ESRD), responsible for about 50% of all cases in the developed countries.^1^ Pakistan is the fourth country with the highest global burden of Diabetes mellitus (DM) having 34.5 million diabetics.^2^ The prevalence of chronic kidney disease (CKD) in Pakistan is 21.2% and diabetic nephropathy is the leading cause accounting for 27.1% of all cases.^3^

Globally, CKD remains a major cause of morbidity and mortality, with diabetic nephropathy contributing to nearly one third of CKD-related disability-adjusted life-years.^4^ Renal transplantation markedly reduces mortality compared with dialysis. Wolfe et al. demonstrated a 48–82% reduction in long-term mortality among transplant recipients, including a 73% reduction in mortality among patients with ESRD due to DM.^5^ Therefore, renal transplantation is the optimal treatment modality for ESRD.

Despite the survival benefits, only a minority of patients with diabetes and ESRD are considered for renal transplantation.^6^ Data on patient survival following renal transplantation are conflicting. Some studies reported decreased survival in diabetic recipients compared with non-diabetic recipients.^7^-^11^ While, other studies have demonstrated comparable survival rates in both recipients.^6^,^12^,^13^ Post-transplant cardiovascular events and infections were found to be remarkably more prevalent and deadly in recipients with diabetes than non-diabetic recipients.^7^,^10^-^14^

Data on kidney transplant recipient survival and post-transplant complications in diabetic recipients are lacking in Pakistan, where the high national diabetes burden, healthcare infrastructure, and socioeconomic barriers may influence transplant outcomes differently than in high-resource settings. To address this gap, this study investigated the influence of recipients’ pre-transplant diabetes on the outcomes of living donor kidney transplantation.

## METHODOLOGY

This prospective observational cohort study was conducted at Dow University Hospital in Karachi, Pakistan. After obtaining informed consent from all study participants, 214 patients aged ≥ 18 years of both sexes who underwent living donor kidney transplantation between September 2022 and December 2023 were recruited using a non-probability consecutive sampling technique. We excluded 12 cases of new-onset diabetes after transplantation (NODAT) to isolate the effects of pre-transplant diabetes on renal transplant outcomes and included 202 patients, who were stratified into two groups based on the presence or absence of pre-transplant DM. The groups were not matched for baseline clinical characteristics.

The total sample size of 172 was derived from the PASS software by taking the prevalence of survival, P_1_ = 75% in the DM group and P_2_ = 90.8% in the non-DM group, using 80% power of the test and 95% confidence level.^11^ However, we initially enrolled 214 patients to account for possible dropouts. All transplant recipients were administered induction therapy with either anti-thymocyte globulin or basiliximab based on their risk profile. Mycophenolic acid, prednisolone, and tacrolimus or cyclosporine were used in the maintenance immunosuppressant regimen.

Demographic, clinical, and laboratory data were collected. Study participants were followed up monthly for one year following kidney transplantation at the renal transplant clinic and observed for one-year patient survival and post-transplant complications.

### Ethical approval:

This study was conducted after obtaining approval from the Institutional Review Board (reference number: IRB-3087/DUHS/EXEMPTION/2023/296; dated: 03/08/2023).

### Exclusion criteria:

- History of malignancy before kidney transplantation
- Patients having NODAT
- Non-compliant to immunosuppressant medication following kidney transplantation
- Patients having irregular follow-ups or lost to follow-up

### Study outcomes:

The primary outcome was one-year patient survival after kidney transplantation. The secondary outcome was post-transplant complications occurring during the first year, including surgical wound dehiscence, delayed graft function, graft rejection, graft failure, infection, cardiovascular events and malignancy.

### Operational definition:

- ***Pre-existing diabetes mellitus:*** Diagnosis of diabetes before kidney transplantation, defined according to the American Diabetes Association criteria as HbA1c ≥ 6.5% or fasting blood sugar (FBS) ≥ 126 mg/dl or random blood sugar ≥ 200 mg/dl in the presence of symptoms of hyperglycemia.
- ***Delayed graft function:*** Defined as the need for dialysis within seven days following kidney transplantation due to insufficient graft function.^15^
- ***Graft rejection:*** Biopsy-proven evidence of rejection based on the Banff Classification system for reporting renal pathology.
- ***Graft failure:*** Kidney transplant recipients requiring chronic dialysis or re-transplantation due to chronic graft dysfunction.

### Statistical analysis:

Statistical analyses were performed using SPSS software (version 27). Continuous variables were compared using the Mann-Whitney U test and are presented as median with interquartile ranges (IQR). Categorical variables were compared using the Chi-square or Fisher’s exact test and are presented as counts with percentages (%). Survival analysis was conducted using the Kaplan–Meier method to compare recipient survival, and the log-rank test was applied to calculate statistical significance. Cox proportional hazards regression analysis was performed to identify independent risk factors for mortality. Univariate analysis was performed to assess the individual effects of each of the covariates. Multivariate analysis of covariates with p-value ≤ 0.05 on univariate analysis was carried out to identify independent predictors of mortality after adjusting for potential confounders such as age and comorbidities due to unmatched cohorts, and results were described as Hazard Ratios (HR) with 95% Confidence Intervals (CI). A p-value of ≤ 0.05 was considered statistically significant.

## RESULTS

Among the 202 kidney transplant recipients, 101 had pre-existing diabetes and 101 were non-diabetic. Males were predominant in both groups. Recipients with pre-existing diabetes were notably older than non-diabetic recipients. The demographic and clinical characteristics are shown in Table-I. Complications were more marked in the pre-existing diabetes group than in the non-DM group (57.4% vs 28.7%, p < 0.001). The details of the complications are presented in Table-II.

**Table-I:** Comparative analysis of the baseline characteristics of Pre-existing Diabetes and Non-DM kidney transplant recipients.

Characteristics	Non-DM group (n=101)	Pre-existing Diabetes group (n=101)	P-value
**Recipient age median (IQR)**	32 (26.5 - 42)	47 (38 - 52)	< 0.001^ꝭ^*
**Recipient gender (%)**			
Male	87 (86.1%)	92 (91.1%)	0.268^χ^
Female	14 (13.9%)	9 (8.9%)	
**Pre-transplant comorbidities (%)**			
Hypertension	101 (100%)	81 (80.2%)	<0.001 ^χ^*
Ischemic heart disease	3 (2.97%)	7 (6.9%)	0.331 ^χ^
Congestive heart failure	0 (0)	1 (0.99%)	1 ^ƒ^
Peripheral vascular disease	0 (0)	3 (2.97%)	0.246 ^ƒ^
Stroke	0 (0)	1 (0.99%)	1 ^ƒ^
Chronic liver disease	0 (0)	1 (0.99%)	1 ^ƒ^
**Number of Human leukocyte antigen match median (IQR)**	3 (3-6)	3 (3-6)	0.291 ^ꝭ^
**Panel reactive antibody (%)**			
Negative	46 (45.5%)	59 (58.4%)	0.067 ^χ^
Positive	55 (54.5%)	42 (41.6%)
**Complement dependent cytotoxicity cross match (%)**			
Negative	100 (99%)	101 (100%)	1^ƒ^
Positive	1 (1%)	0 (0)	
**Pre-transplant FBS median (IQR)**	83 (77 - 92)	135 (107 - 168)	< 0.001 ^ꝭ^*
**Post-transplant FBS median (IQR)**	97 (91 - 104)	153 (137 - 176)	< 0.001 ^ꝭ^*
**Donor age median (IQR)**	32 (24.5 - 40)	32 (26 - 42.5)	0.641 ^ꝭ^
**Donor gender (%)**			
Male	49 (48.5%)	46 (45.5%)	0.672 ^χ^
Female	52 (51.5%)	55 (54.5%)	

*: p-value ≤ 0.05 considered as statistically significant, ꝭ: Mann Whitney U test, χ: Chi-square test, ƒ: Fisher Exact test

**Table-II:** Comparison of Post-transplant complications between the Pre-existing Diabetes and Non-DM kidney transplant recipients

Post-transplant complications	Non-DM group (n=101)	Pre-existing Diabetes group (n=101)	P-value
Delayed graft function	0 (0)	2 (1.98%)	0.498
Graft rejection	17 (16.8%)	16 (15.8%)	1
Graft failure	5 (5%)	6 (5.94%)	1
Surgical wound dehiscence	1 (1%)	2 (1.98%)	1
Infection	23 (22.8%)	47 (46.5%)	< 0.001*
Cardiovascular events	0 (0)	7 (6.9%)	0.014*
Malignancy	0 (0)	1 (0.99%)	1

*: p-value ≤ 0.05 considered as statistically significant

One-year patient survival was 88.1% in the pre-existing DM group compared to 95% in the non-DM group, with a p-value of 0.075, which was not statistically significant, although a numerical trend was observed in the survival rates. Patient survival in both groups is depicted in the Kaplan-Meier curve (Fig.1). Mortality was 11.9% in the pre-existing DM group versus 5% in the non-DM group (p= 0.076).

**Fig.1:**
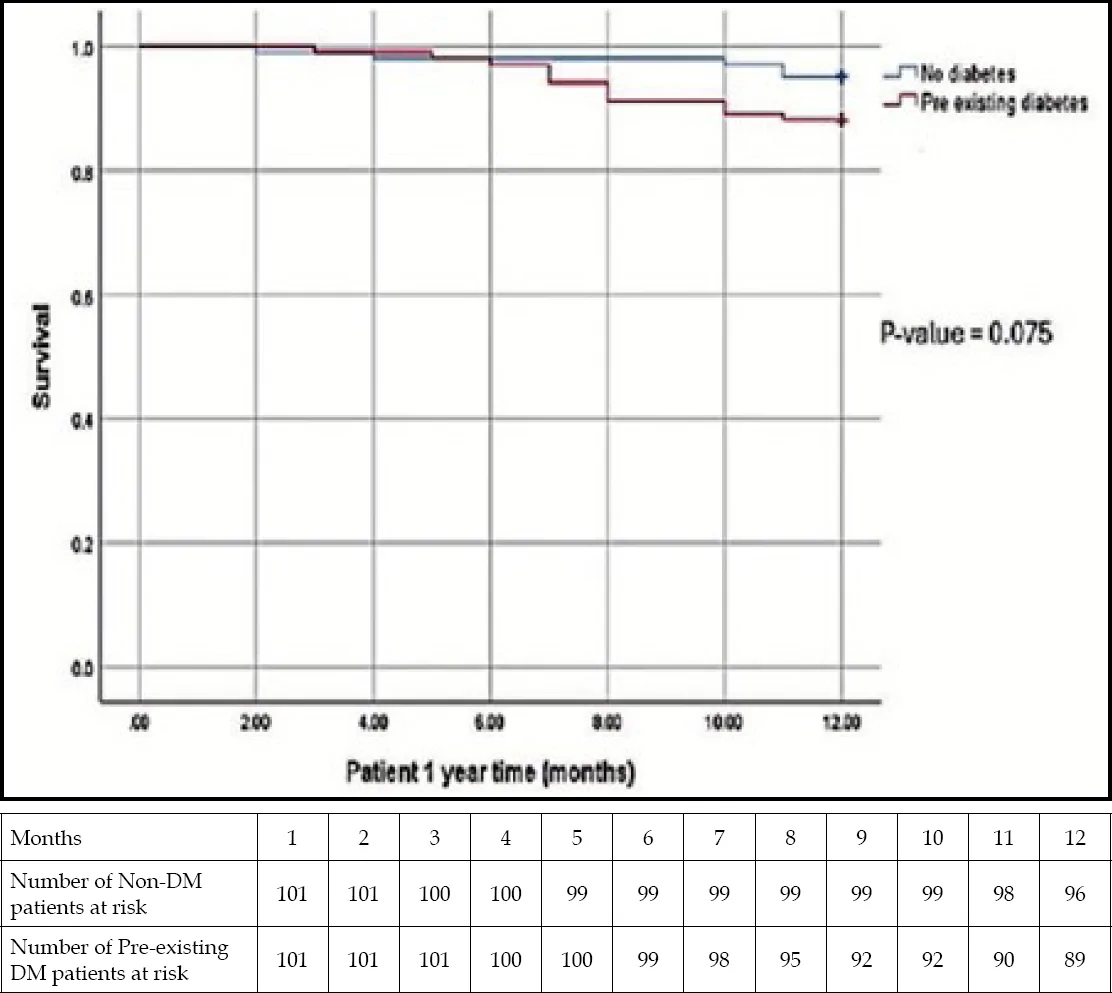
Kaplan Meier survival curves of renal transplant recipients with and without Pre-existing Diabetes

Univariate analysis identified recipient age, pre-transplant cardiovascular comorbidities such as congestive heart failure (CHF), peripheral vascular disease (PVD), ischemic heart disease (IHD), post-transplant cardiovascular events, and elevated pre- and post-transplant FBS levels as potent risk factors for mortality. Pre-existing diabetes was associated with a non-significant hazard of higher mortality.

In the multivariate Cox model, pre-existing diabetes was paradoxically associated with a lower hazard of mortality after adjusting for age, pre-transplant CHF, pre-transplant PVD, pre-transplant IHD, post-transplant cardiovascular events, and pre- and post-transplant FBS, which is a statistical artifact rather than genuine protective effect. Pre-transplant CHF, pre-transplant PVD, post-transplant cardiovascular events, and elevated post-transplant FBS levels were independent predictors of mortality (Table-III).

**Table-III:** Cox proportional hazards regression analysis of Mortality

	Univariate			Multivariate		
Covariate	HR	95% C.I	P-value	HR	95% C.I	P-value
Pre-existing Diabetes	2.488	0.877---7.064	0.087	0.074	0.009---0.611	0.016 ^†^
Recipient Age	1.074	1.021---1.131	0.006*	1.043	0.976---1.116	0.212
Recipient Gender	1.79	0.514---6.228	0.36	-	-	-
Hypertension	1.796	0.238---13.541	0.57	-	-	-
Pre-transplant Ischemic heart disease	4.397	1.263---15.305	0.020*	4.125	0.833---20.422	0.083
Pre-transplant Congestive heart failure	28.266	3.478---229.740	0.002*	99.431	7.709---128.441	<0.001*
Pre-transplant Peripheral vascular disease	11.486	2.589---50.948	0.001*	7.787	1.197---50.682	0.032*
Donor Age	1.026	0.985---1.068	0.216	-	-	-
Donor Gender	2.206	0.777---6.261	0.137	-	-	-
Human Leukocyte Antigen match	1.018	0.769---1.300	0.889	-	-	-
Panel Reactive Antibody status	0.744	0.283---1.955	0.549	-	-	-
Graft rejection	2.2	0.775---6.245	0.139	-	-	-
Graft failure	1.043	0.138---7.866	0.967	-	-	-
Surgical wound dehiscence	4.258	0.564---32.133	0.16	-	-	-
Post-transplant cardiovascular events	7.347	2.101---25.695	0.002*	7.839	1.423---43.198	0.018*
Pre-transplant FBS	1.016	1.004---1.027	0.007*	1.018	0.994---1.042	0.145
Post-transplant FBS	1.024	1.011---1.037	<0.001*	1.023	1.001---1.046	0.040*

*: p-value ≤ 0.05 considered as statistically significant

†: statistical artifact due to over-adjustment for cardiovascular mediators, collinearity, and model instability

## DISCUSSION

This study revealed a slightly lower one-year patient survival after kidney transplantation in recipients with pre-existing diabetes than in non-diabetic recipients (88.1% vs. 95.0%), with a p-value that was not statistically significant (p = 0.075). This pattern is concordant with that of Maamoun et al., who reported 82% versus 89% one-year post-transplant survival in patients with DM and without DM, respectively.^13^ Although numerical trends suggested a possible early survival disadvantage in diabetic recipients, one-year survival outcomes remained statistically comparable in our study, which may reflect the mitigating effects of restored renal function and reduced metabolic stress following transplantation.

Jeon et al. identified diabetes as an independent predictor of mortality after adjusting confounders.^10^ In contrast, the paradoxical inverse association between pre-existing diabetes and mortality observed in our multivariate model is most plausibly explained as a statistical artifact rather than a true protective effect. This likely reflects over-adjustment for cardiovascular mediators, collinearity, and model instability due to the limited number of deaths in the study.

In this prospective study, elevated median post-transplant FBS, derived from serial readings per recipient, showed a modest but independent association with mortality. This limited effect likely reflects the inability of FBS values alone to capture the overall glycemic burden, as well as the small number of deaths and the stronger impact of comorbid predictors, such as peripheral vascular disease, congestive heart failure, and post-transplant cardiovascular events. Although HbA1c is unreliable in the immediate post-transplant period, it could have provided a more stable measure of long-term glycemic control beyond three months post-surgery. In contrast, Taber et al. found no association between glycemic control and renal transplant recipients’ survival in a retrospective analysis.^8^

In concordance with Cosio et al., pre-transplant CHF, pre-transplant PVD, and post-transplant cardiovascular events were strong independent predictors of mortality in our study, although wide confidence intervals reflect limited precision due to low mortality rates.^7^ Similar to Jeon et al. and Baek et al., we found a heightened incidence of cardiovascular events following transplantation in the pre-existing diabetes group.^10^,^16^ This is attributable to the mechanisms described in literature, resulting from chronic hyperglycemia such as endothelial dysfunction, proinflammatory and prothrombotic states, impaired fibrinolysis and immunosuppressant-related dyslipidemia.^17^,^18^

Consistent with other studies, we observed an increased incidence of infection in the pre-existing diabetes group.^14^,^19^ Chronic hyperglycemia impairs neutrophil and macrophage function, advanced glycation end products disrupt complement activation and cytokine signaling, and microvascular disease compromises tissue perfusion and wound healing. These defects, compounded by immunosuppressive therapy, increase susceptibility to infections.^20^,^21^ This provides a pathological basis for the higher infection burden in the diabetic recipients in this study.

We did not find a significant difference in graft failure in either group, implying that diabetes does not influence graft survival, which concurs with the findings of Jeon et al.^10^ Similar to other studies, we observed analogous graft rejection rates in both groups, indicating that diabetes does not confer an increased risk of graft rejection.^8^,^13^ We noticed a low incidence (1.98%) of delayed graft function (DGF), which occurred only in the pre-existing diabetes group. Yao et al. identified diabetes as the strongest independent risk factor for DGF (HR 1.52, [95% CI: 1.40–1.64, p < 0.0001]).^22^ Delayed graft function did not affect patient survival in the current study, although a profound effect on patient survival was shown by Cosio et al.^7^ The low incidence of DGF in the present study was possibly due to the utilization of living donor kidney transplants and the younger age of the donors.

This is the first prospective study in Pakistan. It provides region-specific evidence to guide clinical decision making. Diabetic recipients, owing to their high-risk profile, represent a vulnerable population for post-transplant cardiovascular events and infections. This study highlights the need to improve current protocols for screening and treating CVD before transplantation in recipients with DM. Implementing infection control strategies, ongoing cardiovascular surveillance, and strict glycemic control after transplantation is essential to minimize the risk of life-threatening complications. This study supports expanding transplant access for patients with DM and ESRD, particularly for carefully selected patients with DM in high-burden regions after thorough pre-transplant cardiovascular screening, where exclusion based solely on glycemic status may be unjustifiable. Given the paucity of region-specific data, we suggest a multicenter longitudinal study in Pakistan to explore the long-term effects of diabetes on kidney transplant outcomes.

### Strengths:

The strengths of this study include its prospective design, consecutive recruitment, and systematic outcome ascertainment, which enhance its validity. Multivariate Cox regression enabled adjustment for major comorbidities.

### Limitations:

This was a single-center study with unmatched cohorts, exclusion of NODAT cases, short duration of follow-up, and reliance on median FBS rather than HbA1c levels. The small number of deaths led to wide confidence intervals, limited precision, and an unstable model for the analysis. Over-adjustment and collinearity further contributed to the paradoxical association between DM and mortality.

## CONCLUSION

Despite the increased incidence of post-transplant complications, pre-existing diabetes did not influence one-year transplant recipient survival or renal graft integrity. Pre-transplant CVD and post-transplant cardiovascular events were the strongest independent predictors of mortality in kidney transplant recipients. Rigorous peri-transplant cardiovascular assessment and management, along with stringent post-transplant glycemic control and infection prevention protocols, are warranted to improve post-transplant outcomes.

### Author’s contribution:

**HS:** Designed the study, collected and analyzed the data, interpreted the results, performed literature search, and prepared the manuscript.

**ZK** and **MTK:** Critically reviewed the manuscript for intellectual content.

**AR:** Analyzed data, interpreted the results and critically reviewed the manuscript.

All authors have read and approved the final manuscript and have taken responsibility for its accuracy and integrity.
